# Hypoxia in the Baltic Sea: Biogeochemical Cycles, Benthic Fauna, and Management

**DOI:** 10.1007/s13280-013-0474-7

**Published:** 2014-01-12

**Authors:** Jacob Carstensen, Daniel J. Conley, Erik Bonsdorff, Bo G. Gustafsson, Susanna Hietanen, Urzsula Janas, Tom Jilbert, Alexey Maximov, Alf Norkko, Joanna Norkko, Daniel C. Reed, Caroline P. Slomp, Karen Timmermann, Maren Voss

**Affiliations:** 1 Department of Bioscience, Aarhus University, Frederiksborgvej 399, 4000 Roskilde, Denmark; 2 GeoBiosphere Science Centre, Department of Geology, Lund University, Sölvegatan 12, 223 62 Lund, Sweden; 3 Department of Biosciences, Environmental and Marine Biology, Åbo Akademi University, 20500 Turku, Finland; 4 Baltic Nest Institute, Stockholm University, 106 91 Stockholm, Sweden; 5 Department of Environmental Sciences, Aquatic Sciences, University of Helsinki, PO BOX 65, 00014 Helsinki, Finland; 6 Institute of Oceanography, University of Gdansk, al. Marszałka J. Piłsudskiego 46, 81-378 Gdynia, Poland; 7 Faculty of Geosciences, Utrecht University, Budapestlaan 4, 3584 CD Utrecht, The Netherlands; 8 Zoological Institute, Russian Academy of Sciences, Universitetskaya nab. 1, 199034 St. Petersburg, Russia; 9 Tvärminne Zoological Station, University of Helsinki, J.A. Palméns väg 2600, 10900 Hanko, Finland; 10 Leibniz-Institute of Baltic Sea Research, IOW, Seestr. 15, 18119 Rostock, Germany

**Keywords:** Climate change, Ecosystem recovery, Ecosystem services, Eutrophication, Nutrient management, Regime shift

## Abstract

Hypoxia has occurred intermittently over the Holocene in the Baltic Sea, but the recent expansion from less than 10 000 km^2^ before 1950 to >60 000 km^2^ since 2000 is mainly caused by enhanced nutrient inputs from land and atmosphere. With worsening hypoxia, the role of sediments changes from nitrogen removal to nitrogen release as ammonium. At present, denitrification in the water column and sediments is equally important. Phosphorus is currently buried in sediments mainly in organic form, with an additional contribution of reduced Fe-phosphate minerals in the deep anoxic basins. Upon the transition to oxic conditions, a significant proportion of the organic phosphorus will be remineralized, with the phosphorus then being bound to iron oxides. This iron-oxide bound phosphorus is readily released to the water column upon the onset of hypoxia again. Important ecosystems services carried out by the benthic fauna, including biogeochemical feedback-loops and biomass production, are also lost with hypoxia. The results provide quantitative knowledge of nutrient release and recycling processes under various environmental conditions in support of decision support tools underlying the Baltic Sea Action Plan.

## Introduction

Over the twentieth century nutrient inputs to the Baltic Sea increased by factors of three and five for nitrogen and phosphorus, respectively, with widespread eutrophication as a consequence (Gustafsson et al. 2012). One of the most deleterious effects of eutrophication is the increase in hypoxia (Österblom et al. 2007; Zillén and Conley 2010; HELCOM 2013), here defined as oxygen concentrations less than 2 mg L^−1^. Bottom-water oxygen concentrations are also modulated by physical factors, particularly the frequency and intensity of inflow of saltier water, which is governed by meteorological forcing and varies over decades (Meier 2007; Reissmann et al. 2009). Salt water inflows often bring new supplies of oxygen to bottom waters, but at the same time enhance stratification and thereby reduce the vertical mixing of oxygen across the halocline. During the stagnation period from 1983 to 1993, which was characterized by a lack of major Baltic inflows (MBI) (Matthäus et al. 2008), the extent of hypoxia was more than halved (Conley et al. 2002).

Hypoxia influences nutrient removal processes directly through changing the cycling of N and P compounds and indirectly through eradicating the benthic community, which normally enhances biogeochemical processes through bioturbation and bioirrigation. Nutrient releases from sediments under hypoxic conditions can be substantial. For example, phosphorus mobilization can exceed the land-based loading by factors up to three (Conley et al. 2002). Over time this additional internal phosphorus release decreases the N/P ratio in the surface layer favoring nitrogen-fixing cyanobacteria, a prominent feature of the Baltic Sea. The significant biomass of cyanobacteria further enhances the vertical flux of organic material to bottom waters and stimulates further aerobic respiration. Thus, there are important feedbacks between hypoxia and biogeochemical cycles that may sustain the so-called “vicious cycle of the Baltic Sea” (Vahtera et al. 2007). Although our conceptual understanding of these processes is well-developed, there are still gaps in our quantitative understanding of the relative importance of various processes, particularly the influence of benthic fauna in modulating process rates.

The Helsinki Commission (HELCOM) has formulated five ecological objectives for combating eutrophication; one of these is to establish natural oxygen levels. Since hypoxia-induced nutrient releases from the sediments can be substantial and reach the surface layer, the four other ecological objectives (natural levels of nutrients, clear water, natural level of algae blooms, and natural distribution and occurrence of benthic plants and animals) are indirectly linked to the oxygen objective and can be fulfilled only if oxygen conditions improve. Nutrient reductions to achieve these ecological objectives constitute an important component of the Baltic Sea Action Plan (BSAP; HELCOM 2007). The decision support tools underlying the BSAP have, however, lacked detailed quantitative knowledge of nutrient release and recycling processes under various environmental conditions. Thus, an improved description of the internal nutrient loading from the sediments will improve estimates of nutrient reductions required to achieve the desired ecological objectives.

In this paper, we review our current understanding of factors governing hypoxia in the Baltic Sea and specifically demonstrate how results from the HYPoxia mitigation for Baltic Sea Ecosystem Restoration (HYPER) project have contributed to advance this. The HYPER project followed the concept “understanding the past to model the present and predict the future” with specific objectives: (1) to improve understanding of historical trends of hypoxia in relation to physical and climatic variation, (2) to understand and quantify the relationships between oxygen concentrations, benthic organisms and biogeochemical processes, (3) to obtain a better spatial description of the biogeochemical processes that improves upscaling to entire basins, and (4) to improve current decision support tools for the BSAP (HELCOM 2007). First, we describe the long-term variations in oxygen conditions. Second, we review the current understanding of how hypoxia affects the cycling of nitrogen and phosphorus, and how the benthic fauna modulates these processes. Finally, we address the implications on ecosystem services based on the improved knowledge obtained in HYPER.

## Trends of Hypoxia Over the Holocene and Anthropocene

### Paleoindicators of Hypoxia

The seminal paper by Zillén et al. (2008) demonstrated that consistent patterns in hypoxia through time were observed throughout the Baltic Proper. Three defined intervals of frequent hypoxia have been identified during the past ca. 8000 years in the Baltic Sea. The first hypoxic events were dated to around 7000–4000 B.P., in the part of the Early Holocene known as the Littorina transgression. This interval followed the seawater intrusion through the Danish straits due to eustatic sea level rise, which transformed the freshwater Ancylus Lake to the brackish Littorina Sea. This intrusion of seawater increased the stratification of the water column of the Baltic, and has been hypothesized to be the primary cause of deep-water hypoxia (Zillén et al. 2008). Around 4000 B.P. the Littorina Sea stabilized, and salinity decreased due to the reduction in size of the Danish straits, resulting in increased vertical mixing and reoxygenation of the deep basins.

Hypoxia was again observed ca. 1000–700 B.P. during the Medieval Climate Anomaly (MCA). Two important factors may have contributed to hypoxia during this interval. First, Northern Europe experienced milder winters due to a persistently positive phase of the North Atlantic Oscillation (NAO) climate mode. Second, the population for many of the countries in the Baltic Sea watershed nearly doubled within 300 years (Zillén and Conley 2010), leading to land use changes and increased terrestrial nutrient runoff. Hypoxia disappeared during the Little Ice Age (1350–1850), as the NAO shifted to a more persistently negative phase, leading to an increase in storm frequency and enhanced mixing of the water column (Kabel et al. 2012). In addition, population decreased during the fourteenth century when the Black Death and famine hit Europe. The onset of modern hypoxic conditions in the last half of the twentieth century to present is directly linked to excess nutrient loading from agricultural activities and urban development in the past century (Conley et al. 2002).

**Fig. 1:**
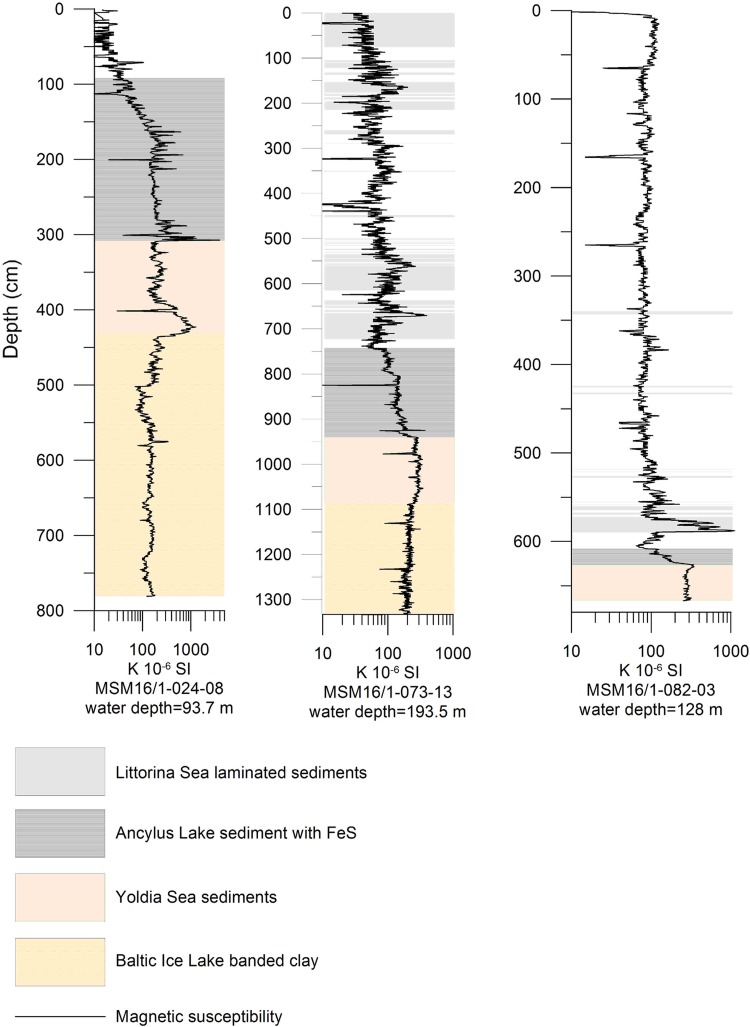
Spatial variability in sediment laminations, an indicator of hypoxia (Zillén et al. 2008). The long sediment cores contain three different periods of Baltic Sea history including the Baltic Ice Lake, the Ancylus Lake, and the Littorina Sea. High values of magnetic susceptibility during laminated periods are due to the occurrence of magnetic gregite deposited by bacteria (Reinholdsson et al. 2013)

Sediment laminations in Littorina Sea sediments varied spatially (Fig. 1), with none in the Arkona Basin and the Bornholm Basin, widespread occurrences in the Gotland Basin, and presence only during the early Littorina Sea in the Bothnian Sea. It is likely that hypoxia was prevalent for longer periods in the deepest areas of the Baltic Sea, e.g., the Landsort Deep and the deepest areas of the Gotland Basin. However, variations occur in the intensity and distribution of laminated sediments, which remain to be fully explained. Brief periods (a few years to a few decades) with oxic conditions punctuated the anoxic background conditions and permitted a restricted benthic community, which mixed the sediments through their burrowing activity. These observations imply more dynamic and oxic conditions in the Gotland Deep than previously thought (Virtasalo et al. 2011).

### Historic Water Quality Data

Although bottom waters may have changed from oxic to anoxic conditions several times during the Holocene, the rate of change in oxygen concentrations and the expansion of hypoxia over the last 100 years has been unprecedented (Zillén and Conley 2010). Dissolved oxygen concentrations in the water column were first measured around 1900 during different research cruises and sporadically in time and space. Monitoring programs were established in the 1960s and 1970s with regular sampling that allowed for assessing volume and areal extent of hypoxia (Karlson et al. 2002). The data from 1960 to present do not yield a significant trend with time (Conley et al. 2009a), because eutrophication and therefore hypoxia was most likely already prominent at the onset of measurements. Significant variations caused by physical factors, such as lack of inflows during the stagnation period (1983–1993), masked any potential trend in response to increasing nutrient inputs. The sporadic data from before 1960 at specific locations, particularly the Gotland Basin, demonstrate declines in oxygen concentrations compared to recent data (Fonselius and Valderama 2003; Gustafsson and Stigebrandt 2007). Increases in the spatial extent of hypoxia are evident from a few specific periods (Savchuk et al. 2008).

**Fig. 2:**
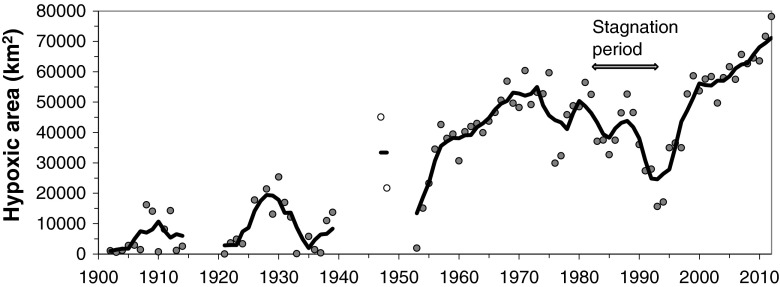
Long-term variations of the bottom area covered with waters containing less than 2 mg L^−1^ oxygen with a 5-year moving average (*solid line*). Estimates from the late 1940s were based on limited data, and are therefore not included in the moving average trend. During the stagnation period (1983–1993) the area of hypoxia decreased

A parametric approach was used to model the vertical profiles for oxygen conditions from 1900 to present (HELCOM 2013), assuming that salinity changes with depth could be described as a sigmoid function of three parameters and that the oxygen profile could be described by two parameters in addition to the information obtained from the salinity profile. This method provided a relatively consistent time series of oxygen conditions, showing that hypoxia was confined to a relatively small area before 1950 (Fig. 2) and then increased to more than 50 000 km^2^ around 1970. During the stagnation period (1983–1993) the hypoxic area was strongly reduced due to weaker stratification, resulting in stronger mixing across the halocline and a deepening of the mixed layer above the halocline by more than 10 m (HELCOM 2013). Enhanced inflows of less dense water interleaving below the halocline may also have contributed to improved oxygen conditions. Since the stagnation period, hypoxia has expanded again to over 60 000 km^2^ in recent years (Fig. 2). The recent expansion can be explained by the stronger stratification and an upward movement of the halocline in the water column. Stratification has intensified since 1993 despite relatively few MBIs over the last couple of decades. HELCOM (2013) also demonstrated that the large change in hypoxia since 1900 was mainly attributable to increasing nutrient inputs to the Baltic Sea.

## Hypoxia and Nitrogen Cycling

Nitrogen limits primary production in most of the Baltic Sea (Kivi et al. 1993; Tamminen and Andersen 2007). High nitrogen availability in spring generates extensive phytoplankton blooms, the sedimentation and consequent mineralization of which leads to oxygen deficiency during seasonal stratification. Low-oxygen conditions below the pycnocline affect nitrogen cycling, with possible positive feedbacks to nitrogen availability in the surface waters.

**Fig. 3:**
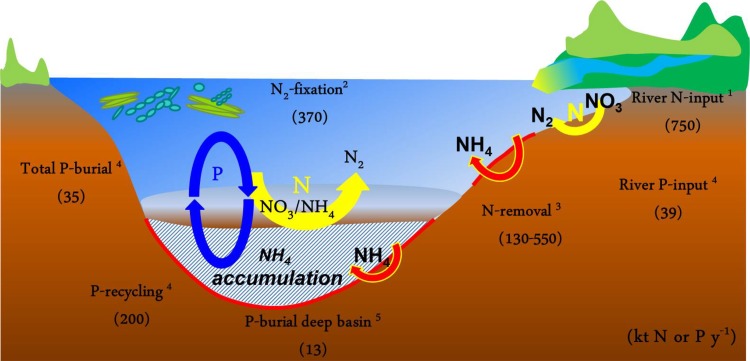
Schematic of Baltic Sea N and P cycling in the basin and at coastal river-impacted sites. *Yellow arrows* indicate denitrification/anammox processes at the redoxcline and in coastal sediments. The *red arrows* indicate the other pathway, which leads to ammonium release and accumulation. *Black numbers* are estimates of nitrogen and phosphorus sources and sinks (references for the different fluxes are (*1*) Voss et al. 2011; (*2*) Wasmund et al. 2001; (*3*) Dalsgaard et al. 2013; (*4*) Gustafsson et al. 2012; (*5*) Mort et al. 2010)

Nitrogen cycling is microbially mediated, and includes multiple processes that lead to formation of N_2_ gas that can escape the water column to the atmosphere (Ward et al. 2007). This natural nitrogen removal is an important ecosystem service mitigating eutrophication. For this sequence to proceed efficiently, both oxic and anoxic conditions are needed. Ammonium released in organic matter mineralization is nitrified in oxic conditions, with the products nitrite and nitrate feeding the anoxic denitrification and anammox processes. These processes usually take place in sediments that typically have oxygen penetration depths of a few mm only, allowing close coupling of oxic and anoxic processes. In the Baltic Sea, denitrification is responsible for most of the nitrogen removal (Fig. 3), with anammox contributing only occasionally (Hietanen 2007; Hietanen and Kuparinen 2008; Jäntti et al. 2011; Dalsgaard et al. 2013).

Short periods of hypoxia and anoxia can enhance the anoxic denitrification process, as long as nitrate is available (Hietanen and Lukkari 2007). However, repeated or prolonged hypoxia results in loss of the oxidized sediment layer supporting denitrification, bringing the hypoxic surface layer in direct contact with reduced, sulfidic sediment. Nitrification, which requires oxygen, is possible only at the sediment surface, below which nitrate can be reduced back to ammonium, instead of N_2_ gas, by dissimilatory nitrate reduction to ammonium (DNRA) powered by H_2_S seeping from the reduced sediment. The switch from N removal by coupled nitrification- denitrification to N storage by coupled nitrification-DNRA takes place at oxygen concentrations around 3.4 mg L^−1^ in the Gulf of Finland (Jäntti and Hietanen 2012). DNRA dominates nitrate reduction at even higher oxygen conditions in seasonally hypoxic coastal areas of the southern Baltic Sea (Dale et al. 2011), which is likely related to the higher mineralization rates and hence higher oxygen demand in those sediments. No DNRA has been detected in northern Baltic coastal areas (Jäntti et al. 2011; Jäntti and Hietanen 2012). However, coastal N removal has also decreased, with rates measured in the northern Gulf of Finland in 2007–2009 being almost 50 % lower than those measured at the same station in 2003–2004 (Hietanen and Kuparinen 2008; Jäntti et al. 2011). This decrease is possibly related to more frequent hypoxic events. Thus, hypoxia alters the pathways of nitrogen cycling at higher oxygen concentrations than previously expected, when oxygen disturbance takes place frequently or for long time period.

When complete anoxia sets in, nitrification ceases. Nitrogen removal cannot proceed due to lack of nitrate. The coupled nitrification-DNRA nitrogen storage, prevailing in hypoxic conditions, fails, and sediments become sources of ammonium. This has already happened in the deepest, central areas of the Gulf of Finland. In the mid-1990s these areas were oxic and supported active denitrification (Tuominen et al. 1998). In 2008, resampling of the same stations returned only sulfidic sediments with no nitrogen retained or removed (Jäntti and Hietanen 2012).

In addition to seasonal hypoxia and anoxia, large areas of the Baltic Sea suffer from permanent anoxia. Active nitrification takes place at the interface between the oxic surface layer and anoxic, ammonium-rich water below the redoxcline, located near the halocline (Hietanen et al. 2012). Natural abundance data of stable isotopes in nitrate and nitrite suggest that nitrate generated in these layers is immediately reduced to N_2_ gas. Deeper waters harbor a chemolithotrophic denitrifying community, capable of reducing nitrate to N_2_ gas by using H_2_S as an energy source (Brettar and Rheinheimer 1991). It has been shown that denitrification occurs in a relatively thin and unstable layer of only 3–6 m below the halocline, where nitrate is still present and oxygen is sufficiently low (Dalsgaard et al. 2013). Moreover, irregular large-scale mixing events may generate thicker layers with low-oxygen and nitrate in which large quantities of nitrate are converted to N_2_ gas (Dalsgaard et al. 2013). These conditions can lead to extremely high nitrogen removal rates in the water column and play a significant role for overall N removal (Hietanen et al. 2012; Dalsgaard et al. 2013). While denitrification rates in the sediments in shallow, oxic areas are lower than the potential rates in the water column, they are more constant over time, highlighting the need to abate hypoxia to improve N removal (Hietanen et al. 2012).

## Hypoxia and Phosphorus Cycling

Phosphorus (P) loads to the Baltic Sea increased fivefold over the twentieth century, reaching peak values of 75 kton year^−1^ around 1980 (Gustafsson et al. 2012). Although loads then gradually decreased, surface concentrations of phosphate in the water column in the Baltic Proper and Gulfs of Finland and Riga continued to rise (Gustafsson et al. 2012). This lack of a response to a load reduction is the result of both the long residence time of P in the Baltic Sea and its strong internal recycling (Conley et al. 2002). Results of model simulations with a biogeochemical model of the Baltic Sea (BALTSEM) indicate that increased recycling of P more than compensates for the load reduction and is responsible for the continued eutrophication (Gustafsson et al. 2012).

Various recent field and modeling studies of sediment P dynamics in the Baltic proper and Gulf of Finland confirm the enhanced release of P from anoxic and hypoxic sediments and provide insight into the relevant mechanisms. For example, the highest sediment–water exchange rates of P are observed at sites that have recently undergone a redox change from oxic to anoxic conditions. This is the direct result of reductive dissolution of Fe (oxyhydr)-oxides (henceforth termed Fe-oxides) upon the onset of hypoxia and release of the associated P (Mort et al. 2010; Jilbert et al. 2011; Reed et al. 2011). Preferential regeneration of P relative to C (and N) from organic matter in the water column and sediment is also important and helps support a continued return flux of P from the deep basins to the surface layer (Jilbert et al. 2011). While this enhanced regeneration has been observed previously in low-oxygen marine systems (e.g., Ingall and Jahnke 1994), the mechanism has remained unclear. Recent work by Steenbergh et al. (2013) shows that the stoichiometry of the microbes involved in organic matter breakdown in the sediments plays a key role. At ca. 400:1, the C:P ratio of prokaryotes in surface sediments of the Baltic Sea is higher than the Redfield ratio for marine organic matter of 106:1. This implies that the microbes have no need for all the P in the organic matter to build their cells, explaining why they allow a major proportion of the P to escape to the overlying water under anoxic conditions. Microbes in Baltic Sea surface sediments also have been shown to be C-limited (Steenbergh et al. 2011). They additionally produce abundant enzymes to cleave P from organic matter (phosphatases), likely with the purpose of making the remaining organic matter more accessible for degradation.

**Fig. 4:**
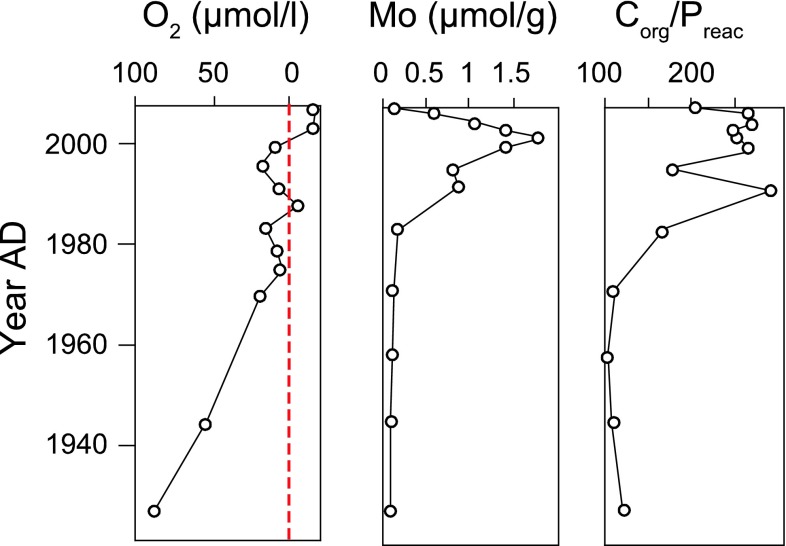
Trends of bottom-water oxygen concentrations in the Northern Gotland basin in the late twentieth century (*left*), and corresponding sedimentary proxies for the intensity of hypoxia; molybdenum concentrations (*center*) and organic carbon:reactive phosphorus ratios (*right*); data from Mort et al. (2010). Note the reversed scale for oxygen. Hypoxia is defined as oxygen concentrations <62.5 μmol L^−1^ (~2 mg L^−1^)

Enhanced regeneration of P relative to C under anoxic conditions is also reflected in sediment records of the ratio of organic C to reactive P (potentially biologically available P). This ratio increases with a decline in bottom-water oxygen and rise in bottom-water sulfide for which sediment molybdenum (Mo) is a proxy (Fig. 4). This does not imply that the burial of P declines during anoxic periods, as suggested previously (Conley et al. 2009a). Instead, burial of organic P in the Baltic Sea has increased with expanding anoxia because of the increased input of organic matter to the sediments (Mort et al. 2010; Jilbert et al. 2011). At present, organic P is quantitatively the most abundant burial form of P in recent anoxic Baltic Sea sediments (Mort et al. 2010; Jilbert and Slomp 2013). While there is little formation of authigenic P minerals at oxic and seasonally hypoxic sites, there is significant formation of Mn–Ca-carbonate-P phases in the anoxic and sulfidic (euxinic) deep basins following inflow events of North Sea water. Surprisingly, these sulfide-rich sediments also contain abundant Fe-bound P (Jilbert and Slomp 2013). The Fe(II)-P, which may be vivianite, is suggested to be formed through sulfidization of Fe-oxides containing P that are laterally transferred to these deep basins from surrounding shallower areas. A third authigenic P-bearing mineral, carbonate fluorapatite, is observed in microfossils and either precipitates within these biogenic structures in the water column or at the sediment–water interface. This phase contributes a relatively constant background burial flux of P (Jilbert and Slomp 2013). In the modeling study of Gustafsson et al. (2012), the total burial of P in the Baltic Sea was estimated at 35 kton year^−1^ for the period 1997–2007 (Fig. 3). Extrapolation of burial rates for various sites in the Baltic Proper indicate that a significant proportion of this P, possibly up to 13 kton year^−1^ is being buried in the deep basins of the Baltic Proper, mostly as organic P, but also as Fe(II) bound P (Mort et al. 2010; Jilbert and Slomp 2013).

Forced reoxygenation of the bottom waters in the Baltic Sea has been proposed to mitigate hypoxia in the Baltic Sea (Stigebrandt and Gustafsson 2007). The potential consequences for the P cycle were recently explored with a coupled benthic–pelagic model (Reed et al. 2011). This work showed that forced reoxygenation leads to an efficient conversion of organic phosphorus to Fe-oxide bound phosphorus in the sediment. If the system would become hypoxic again, however, this Fe-oxide bound P would be quickly released to the overlying water. Thus, if Baltic Sea bottom waters are artificially reoxygenated, it is critical that the waters subsequently remain oxic, otherwise conditions will deteriorate instead of improve (Reed et al. 2011). Given current global warming and the large pool of reactive P in the water column and surface sediments of the Baltic Sea, a significant further reduction of external P loads is required to improve water quality in the Baltic Sea over the coming century (Gustafsson et al. 2012; Meier et al. 2011).

## Hypoxia and Benthic Fauna

Benthic macrofauna inhabiting soft sediments plays an important role for the degradation of organic material and nutrient cycling with feedbacks to pelagic productivity (Aller and Aller 1998). Through their particle mixing and irrigation of tubes and burrows, benthic fauna enhances the transport of material and solutes between different redox zones, affecting not only degradation rates and pathways (Nielsen et al. 2004; Papaspyrou et al. 2005), but also the fluxes of oxygen, nutrients and minerals between the sediment and overlying water in a species-specific manner (Karlson et al. 2007; Braeckman et al. 2010). Infauna creates oxic zones around their burrows allowing aerobic processes to occur in otherwise reduced sediments (Wenzhöfer and Glud 2004).

It is well known that severe hypoxia and anoxia change the behavior and physiology, and ultimately kill benthic fauna (Diaz and Rosenberg 2008; Rabalais et al. 2010). Although salinity sets the limits for benthic macrofaunal diversity in the Baltic Sea and the number of functional groups is low (Segerstråle 1957; Bonsdorff and Pearson 1999; Villnäs and Norkko 2011), bottom-water hypoxia is currently the main factor structuring the benthic communities in the Baltic Proper and Gulf of Finland, resulting in large areas completely devoid of macrofauna (Karlson et al. 2002; Conley et al. 2009a; Villnäs and Norkko 2011). Recently, the problem of seasonal hypoxia in shallower, near-shore areas has also become more prominent (Conley et al. 2011), which is of concern, as this is where the greater macrofaunal biomasses usually are observed (Cederwall and Elmgren 1990). Eutrophication generally enhances biomass production at early stages of nutrient loading and organic enrichment (i.e., increased food availability), followed by community impoverishment or complete loss when severe hypoxia and anoxia develop (Pearson and Rosenberg 1978; Cederwall and Elmgren 1980, 1990; Timmermann et al. 2012).

Results of field experiments where hypoxia was artificially induced suggest that benthic communities are more sensitive than previously recognized. Degradation due to hypoxia starts already after a few days of severe hypoxia (Villnäs et al. 2012). Repeated short periods of hypoxia, while not completely eliminating the fauna, will result in successively larger changes in ecosystem functioning, and changes in species, biomass, and abundance, often with threshold-like shifts (Villnäs et al. 2012, 2013). Importantly, while no single function showed significant responses at early phases of hypoxia, analyzing multiple functions in concert showed that ecosystem functionality is significantly disrupted at an early stage (Villnäs et al. 2013). During short-term hypoxia the benthic processes may be dependent on meiofauna, inhabiting only the topmost few mm of the sediment (Arroyo et al. 2012).

Evidence from field surveys suggest that fauna and bioturbation are rapidly reduced across gradients from normoxic to anoxic conditions (Josefson et al. 2012). Importantly, bioturbation is dramatically reduced as illustrated by the bioturbation potential index (BPI, Solan et al. 2004), with a threshold at 1.4–2.9 mg L^−1^, below which it decreases rapidly to zero in anoxic sediments (Josefson et al. 2012). Bioturbation affects the fate of organic matter in a species-specific manner. For example, the deep-burrowing, invasive polychaete species *Marenzelleria* sp. increases burial of phytodetritus, thereby slowing down overall degradation rates, and potentially counteracting hypoxia formation in the bottom water (Josefson et al. 2012). This represents a positive feedback mechanism between fauna and the mitigation of hypoxia. *Marenzelleria* has spread rapidly and is now dominant throughout coastal regions of the Baltic Sea, especially in areas prone to hypoxia (Maximov 2011; Josefson et al. 2012).

Transport-reaction modeling based on field results suggests that *Marenzelleria* through bioturbation and irrigation oxygenates the deeper sediments and therefore has the potential to enhance P retention in sediments. This may alleviate P release from bottom waters that might contribute to surface water eutrophication and hypoxia (Norkko et al. 2012). The model suggests that over time bioirrigation leads to a substantial increase in the iron-bound P content of sediments, while reducing the concentration of labile organic carbon.

Importantly, the modeling results suggest that the positive feedback mechanism is density dependent (Norkko et al. 2012). It has been hypothesized that the increased water N/P ratio in the eastern Gulf of Finland after *Marenzelleria* mass development could be a result of bioirrigation and bioturbation, leading to a reduction of phytoplankton biomass (especially nitrogen-fixing cyanobacteria) and chlorophyll-*a* concentrations (Maximov et al. in press). Experimental field work corroborates the importance of key species; the abundance of relatively large-sized individuals, for example, the bivalves *Macoma balthica* and *Mya arenaria*, may completely dominate nutrient fluxes and play a key role in overall ecosystem functionality (Norkko et al. 2013). Thus, in the low-diversity Baltic Sea, species diversity may not be as important (Törnroos and Bonsdorff 2012), highlighting instead the importance of key species and the relative abundance, biomass and size structure in modifying measures of ecosystem function.

**Fig. 5:**
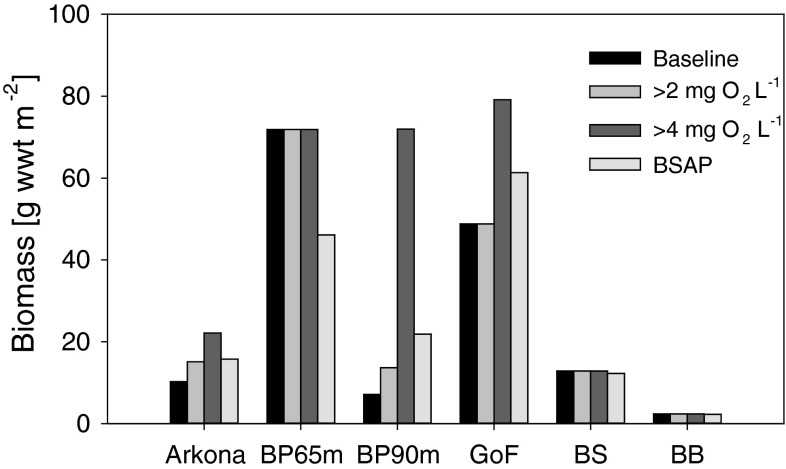
Model-predicted benthic biomass under present (2001–2006) conditions (*baseline*), after introduction of minimum oxygen concentrations of 2 and 4 mg L^−1^, respectively, and after nutrient load reductions according to the Baltic Sea Action Plan (BSAP). Results are shown as 6-year average for monitoring stations in the Arkona basin (Arkona, seasonal hypoxia), a shallow (65 m, normoxic) and deep (90 m, hypoxic) station in the Baltic Proper (BP), in the Gulf of Finland (GoF, occasionally hypoxic), and in the Bothnian Sea (BS, normoxic) and Bothnian Bay (BB, normoxic). The BSAP is expected to increase O_2_ concentrations to >3.5 mg L^−1^ at these six sites. Data from Timmermann et al. (2012)

Karlson et al. (2002) estimated that the benthic biomass missing in the Baltic due to hypoxia/anoxia could be up to 3 million tons, but further suggested that oligotrophication would decrease benthic biomass. However, Timmermann et al. (2012) used a physiological fauna model with five functional groups linked to a three-dimensional coupled hydrodynamic-ecological Baltic Sea model and predicted that benthic biomass would increase sevenfold after re-oxygenating bottom waters (Fig. 5). Modeled nutrient reduction scenarios following the BSAP predicted decreased sedimentation of organic matter of up to 40 % and improved oxygen concentrations in bottom waters. Areas unaffected by hypoxia were predicted to have slight reductions in benthic biomass. As shown by Timmermann et al. (2012), benthic biomass production can increase significantly if oxygen levels are increased not only to 2 mg L^−1^, but rather to 4 mg L^−1^ (Fig. 5). Under such conditions the positive feedback on nutrient dynamics from the benthic communities would help maintain a healthy ecosystem.

Large individuals of long-lived species such as *Macoma* require long periods with suitable oxygen conditions to reach full maturity; only then can efficient positive feedbacks establish (Norkko et al. 2010, 2013). Even short-term repeated hypoxic events can prevent mature communities from developing and severely reduce the resilience of the benthic ecosystem (Villnäs et al. 2013). Recovery of both benthic communities and their functioning after large-scale, long-lasting hypoxia may take several years to decades (Norkko et al. 2010, 2013).

## Hypoxia and Ecosystem Services

An increase in hypoxic events in the Baltic Sea, both intermittent and longer-term over greater geographic areas (Conley et al. 2009a, 2011), has profound impacts on the ecosystem services provided by the benthic ecosystems. In the pelagic realm, fish that either spawn in the hypoxic water layers or demersal-feeding fish suffer losses upon frequent events of oxygen depletion. Furthermore, the biogeochemical processes at the sediment–water interface are directly impacted, which in turn may lead to increased harmful algal blooms, and thus reduced value for recreation and other use of the maritime resources. The direct trophic links from nutrients (primarily flux of P into the water sustaining harmful algal blooms, cyanobacterial blooms, filamentous algal mats) to consumer levels (zoobenthos, fish and even top predators such as birds and mammals, including human consumption) are well known. For management options to be effective there is a need for novel research, illustrating intricate mechanisms that may function as natural remedies for the ecosystem.

It is evident that in order to achieve a balanced ecosystem providing natural goods and services needed, nutrient reductions from land are necessary. Once production levels are slightly reduced, natural processes will enhance the recovery of the system. If, on the other hand, geoengineering in the Baltic Sea is performed without substantial load reductions, the ecosystem services will not recover (Conley et al. 2009b; Conley 2012).
